# Carboxyl Terminus of Hsp70-Interacting Protein Is Increased in Serum and Cerebrospinal Fluid of Patients With Spinocerebellar Ataxia Type 3

**DOI:** 10.3389/fneur.2019.01094

**Published:** 2019-10-15

**Authors:** Zheng-wei Hu, Zhi-hua Yang, Shuo Zhang, Yu-tao Liu, Jing Yang, Yan-lin Wang, Cheng-yuan Mao, Qi-meng Zhang, Chang-he Shi, Yu-ming Xu

**Affiliations:** ^1^Department of Neurology, The First Affiliated Hospital of Zhengzhou University, Zhengzhou University, Zhengzhou, China; ^2^The Academy of Medical Sciences of Zhengzhou University, Zhengzhou University, Zhengzhou, China

**Keywords:** CHIP, SCA3, serum, cerebrospinal fluid, SARA, ICARS, biomarker

## Abstract

**Background:** Spinocerebellar ataxia type 3 (SCA3)/Machado-Joseph disease (MJD) is the most common type of autosomal dominant ataxia. Like other neurodegenerative diseases, is characterized by the dysfunction of the protein quality control (PQC) system. The carboxyl terminus of the Hsp70-interacting protein (CHIP), an important component of PQC, participates in the clearance of misfolded proteins to maintain cellular homeostasis. While no cure for SCA3 exists, the disease progresses slowly. Thus, the identification of biomarkers that indicate the severity and prognosis of this disease would be valuable.

**Methods:** In this exploratory case-control study, we quantitatively evaluated the concentrations of CHIP in the sera of 80 patients with SCA3 and 80 age and sex-matched controls, using the enzyme-linked immunosorbent assay (ELISA). CHIP levels in the cerebrospinal fluid (CSF) donated by six patients and six healthy volunteers, who were matched for sex and age were also measured. All the baseline data were collected, and the patients underwent clinical evaluation. The correlations between CHIP levels and several clinical measurements were analyzed.

**Results:** The serum CHIP level in the SCA3 group was (80.93 ± 28.68) ng/mL, which was significantly higher than those in the control group [(40.37 ± 18.55) ng/mL]. Similar results were observed for the CSF [(164.59 ± 42.99) ng/mL and (37.47 ± 7.85) ng/mL, respectively]. CSF CHIP levels were significantly higher than the serum CHIP levels in the SCA3 group but not in the control group. The Dunn-Bonferroni *post-hoc* for Kruskal-Wallis test revealed no significant difference between the serum and CSF of the patients and the control group. Multivariate linear regression showed that serum CHIP levels correlated positively with disease severity, as measured by the Scale for the Assessment and Rating of Ataxia (SARA) and the International Cooperative Ataxia Rating Scale (ICARS). Moreover, we found that serum CHIP levels were moderately correlated with age in healthy controls.

**Conclusion:** The present study determined that CHIP levels increased significantly in the serum and CSF of patients with SCA3 and that serum CHIP levels were corelated with disease severity. Thus, CHIP is a promising biomarker for SCA3.

## Introduction

Spinocerebellar ataxia type 3 (SCA3), also named Machado-Joseph disease (MJD), is considered to be the most common form of autosomal dominant ataxia (1). The disease is caused by an expansion of the CAG trinucleotide beyond 52 repeats in the *ATXN3* gene (2). The onset of SCA3 is in middle age and it causes a gradual decline in the movement capacity. Other manifestations include dysarthria and dysphagia (3, 4). Similar to other neurogenetic disorders, there is no effective treatment for SCA3 currently. Therefore, monitoring the progress of the disease is important. Biomarkers have important clinical applications in diagnosing and monitoring the disease, as well as evaluating the efficacy of treatments (5). Thus, the identification of biomarkers for SCA3 is essential.

The carboxyl terminus of Hsp70-interacting protein (CHIP), encoded by *STUB1*, is composed of three functional domains: tetratricopeptide repeat (TPR), coiled-coil (CC), and U-box (6). As a component of the E3 ubiquitin ligase, CHIP has been also identified as a dual function cochaperone, because its TPR domain enables binding to heat shock proteins, such as HSP70 and HSP90 (7). The loss of CHIP function is associated with the aggregation of abnormal proteins. Mutations in the *STUB1* gene result in Gordon Holmes syndrome, which is characterized by ataxia and hypogonadism (8, 9). Recent studies have proven that mutation in CHIP could directly cause hereditary ataxia (10, 11).

SCA3 belongs to the class of CAG/polyglutamine repeat diseases. CHIP interacts directly with ataxin-3, a protein encoded by the *ATXN3* gene, which is mainly expressed in the cytoplasm of neurons. Functioning as a deubiquitinase, ataxin-3 regulates the length of the ubiquitin chain of CHIP substrates, as well as the activity of CHIP complexes. PolyQ-expanded ataxin-3 tends to aggregate within the nucleus and forms inclusion bodies, the hallmark of SCA3 (12). Unlike normal ataxin-3, polyQ-expanded ataxin-3 has a higher affinity for CHIP, altering the interaction between the two proteins (13). Research has also shown that CHIP associates with the expanded polyQ protein, and the overexpression of CHIP promotes the degradation of the aggregates (14). Based on these observations, we hypothesized that the serum or cerebrospinal fluid (CSF) of SCA3 patients may exhibit abnormal levels of CHIP.

The present investigation studied a cohort of symptomatic SCA3 patients to demonstrate the potential value of CHIP as a biomarker. The associations among serum CHIP levels, CAG repeat size, age at onset, disease duration, and the Scale for the Assessment and Rating of Ataxia (SARA) and the International Cooperative Ataxia Rating Scale (ICARS) scores were also assessed.

## Materials and Methods

### Participants

This exploratory case-control study was conducted from September 2017 to August 2018. Eighty symptomatic SCA3 patients were recruited from the First Affiliated Hospital of Zhengzhou University. All the patients were adults, who were genetically diagnosed with SCA3 and did not have any other diseases. Eighty age and sex-matched healthy individuals were also included as the control group. A detailed physical examination was performed on healthy controls, including immunological tests and hormone examination, to exclude any diseases. All the participants volunteered to participate in the program.

### Samples Collection and Storage

All participants consented to undergo hemospasia and were instructed to fast before collection of peripheral venous blood. Serum was obtained by blood centrifugation at 3,800 g for 5 min within 1 h of collection, immediately after which the samples were frozen and stored at −80°C. Six patients and six normal volunteers, matched according to age and sex, agreed to donate CSF, which was obtained by lumbar puncture performed under local anesthesia. Individuals who agreed to undergo lumbar puncture were made to lie in a horizontal position, without any pillow after the operation. No side effects were observed. CSF samples were centrifuged to remove cells and precipitates, and the supernatant was stored at −80°C, until analyses were performed.

### Clinical Assessment

Each patient underwent a detailed neurological examination. Basic information was gathered to determine family history, age at onset (AAO), and disease duration (DD). AAO was defined as the age at which symptoms of motor impairment appeared, and disease duration as the interval from AAO to the last hospital visit. SARA and ICARS were used to evaluate disease severity (15, 16). Two experienced neurologists conducted the evaluations separately, and the mean score of the two evaluations was used as the final value.

### Molecular and Biochemical Analysis

Genomic DNA was extracted from peripheral venous blood. After performing the polymerase chain reaction test, capillary electrophoresis was used to determine the number of CAG repeats in the *ATXN3* gene. Levels of CHIP in the serum and CSF were detected with the Human *STUB1* (E3 ubiquitin-protein ligase CHIP) ELISA Kit (#E-EL-H5533c, Elabscience Biotechnology Co., Ltd) according to the manufacturer's instructions. Each sample was measured twice in the same plate to ensure accuracy, and the mean values were used for subsequent data analysis.

### Data Analysis

The raw data were analyzed in the study. The Shapiro-Wilk test was used to assess the normality of the data. The serum CHIP concentration showed an approximately normal distribution. Variance between the case and control groups did not show homogeneity; thus, the corrected Student's *t*-test was used to compare CHIP concentration between the SCA3 and control groups. Comparisons of the serum and CSF CHIP levels between the SCA3 patients and the healthy controls (four groups) were performed with the Kruskal-Wallis test, and Dunn-Bonferroni *post-hoc* were conducted to compare the differences between the specific groups. To explore the associations between CHIP levels and age, number of CAG repeats, AAO, DD, and SARA and ICARS scores, Spearman's correlation tests were performed. Variables that showed a significance of at least 0.2 were incorporated in the stepwise multivariate linear regression model in order to control for confounding factors. SARA score and ICARS score were analyzed separately with other factors in two regression models (Model 1: age, CAG repeats number, AAO, DD, SARA; Model 2: age, CAG repeats number, AAO, DD, ICARS). Pearson's correlation was used to identify the relationship between the serum CHIP level and age for the control group. The relationship between the serum CHIP and gender was analyzed with Spearman's correlation. All the tests were two-sided. Statistical significance was defined as *p* < 0.05. SPSS 21.0 was used for analysis.

## Results

### Increased Serum CHIP Levels in Patients With SCA3

**Figure 1:**
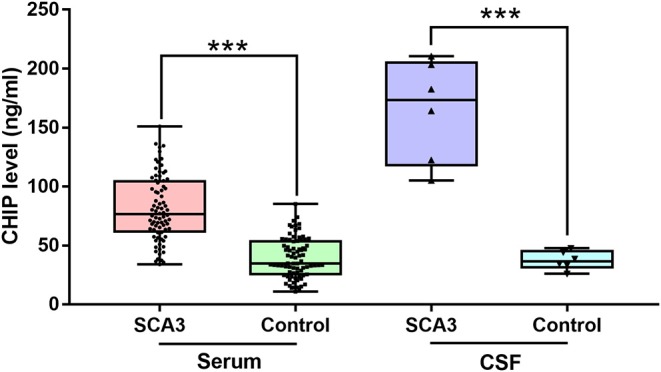
CHIP increased in serum and CSF of patients with SCA3. CHIP level increased in serum and CSF in SCA3 patients significantly. In SCA3 group or healthy controls, no statistical significance was found between serum and CSF CHIP levels. ^***^P < 0.001.

The clinical and molecular data of SCA3 patients and controls were presented as mean ± standard deviation (SD) (Table 1). The mean serum CHIP concentration of the SCA3 group was significantly higher than that of the control group [(80.93 ± 28.68) ng/mL and (40.37 ± 18.55) ng/mL, respectively; *p* < 0.001; 95% CI 27.15–53.94; Figure 1]. In the stepwise multivariate linear regression, the SARA and ICARS scores showed separate correlations in the regression models (SARA: standardized β-coefficient = 0.500, *p* = 0.034; ICARS: standardized β-coefficient = 0.561, *p* = 0.015), while age, AAO, DD, CAG repeats numbers were withdrawn from the model. Correlation analysis revealed a positive correlation between serum CHIP levels and age (*r* = 0.350, *p* = 0.034), but not between serum CHIP levels and sex (*r* = −0.025, *p* = 0.882) in the healthy controls.

**Table 1:** Clinical data statistics and serum CHIP level in SCA3 patients and healthy controls.

**Variables**	**SCA3 patients**	**Controls**
Sex (male/female)	38/42	38/42
Age	50.13 ± 10.47	50.13 ± 10.47
Age at onset (AAO)	42.95 ± 8.83	—
Median of AAO	37.50	—
Disease duration (DD)	7.82 ± 5.48	—
CAG repeats size	65.67 ± 5.05	—
SARA score	9.62 ± 4.93	—
ICARS score	20.03 ± 8.95	—
Serum CHIP level (ng/mL)	80.93 ± 28.68	40.37 ± 18.55

### Increased CSF CHIP Levels in Patients With SCA3

Descriptive statistics were presented as mean ± SD (Table 2). CSF CHIP concentrations were significantly higher in SCA3 patients than those in healthy controls [(164.59 ± 42.99) ng/mL and (37.47 ± 7.85) ng/mL, respectively; *p* < 0.001; 95% CI 82.10–172.13; Figure 1]. On account of the small sample sizes (six patients and six controls), the correlations between CSF CHIP and other impact factors were not analyzed.

**Table 2:** Clinical data statistics and CSF CHIP level in SCA3 patients and healthy controls.

**Variables**	**SCA3 patients**	**Controls**
Sex (male/female)	3/3	3/3
Age	38.83 ± 10.67	38.83 ± 10.67
Age at onset (AAO)	33.17 ± 10.21	—
Median of AAO	32.00	—
Disease duration (DD)	5.67 ± 3.72	—
CAG repeats size	68.50 ± 4.14	—
SARA score	10.42 ± 3.50	—
ICARS score	19.50 ± 5.00	—
Serum CHIP level (ng/mL)	164.59 ± 42.99	37.47 ± 7.85

### Comparison of Serum and CSF CHIP Levels

The Kruskal-Wallis test was performed to compare serum and CSF CHIP levels between patients and controls. Our analyses revealed statistical differences in CHIP levels among the four groups (*p* < 0.001). Dunn-Bonferroni *post-hoc* were conducted to compare differences between specific groups. Results showed no statistical difference between serum and CSF CHIP levels in normal subjects (adjusted *p* = 1.000). However, there was also no statistical difference between serum and CSF CHIP levels, based on the *post-hoc* for Kruskal-Wallis test (adjusted *p* = 0.409) in SCA3 patients.

## Discussion

This study is, to the best of our knowledge, the first to observe increased serum and CSF CHIP levels in SCA3 patients, when compared with healthy participants. Statistical analysis revealed no significance difference between CHIP levels in the CSF and serum of SCA3 patients. Regression analysis showed that serum CHIP levels were significantly associated with SARA and ICARS scores. CHIP levels increased with age in healthy participants. Sex did not have a significant impact in the CHIP levels of the case or control groups.

Molecular biomarkers could be used to track the course of SCAs. In earlier studies, a pilot study showed that neurofilament light (NfL) significantly increased in SCA1, 2, 3, and 6, and could be an indicator of neuronal damage in repeat-expansion SCAs (17). Peripheral reactive oxygen species were considered as potential biomarkers of SCA3 (18). The blood-based transcriptional profile also discovered a pool of up-regulated genes related with the disease (19). Glutathione-S-transferase (GST) activity was found to be higher in SCA2 patients. In a recent study, metabolic profiling revealed perturbed amino acid and fatty acid metabolism in symptomatic SCA3 patients (20). Our results suggest that CHIP could serve as an indicator of the severity of SCA3.

CHIP plays a central role in protein quality control. Since ataxin-3 directly interacts with CHIP and the binding affinity between the two proteins increases when polyglutamine expands, cellular homeostasis is negatively affected in SCA3. In SCA3 transgenic mice, CHIP decreased in brain lysates (13). Abnormally aggregation of proteins eventually leads to cell death. Neurodegeneration occurs in a widespread manner in the central nervous system of patients with SCA3 (21). CSF better reflects the condition of brain diseases, compared to serum. However, due to the limited sample size, the relationship between CSF CHIP levels and clinical parameters was not analyzed. Moreover, the *post-hoc* Kruskal-Wallis test revealed no significant difference between CSF and serum, which suggests that serum CHIP levels could indicate the CSF CHIP levels to some extent.

CHIP is ubiquitously expressed in tissues, similar to ataxin-3 (6, 22). Our data indicate that serum CHIP concentrations are significantly higher in SCA3 patients than those in healthy controls. Multivariate linear regression was performed, to control for confounding factors. The regression equation showed that both the SARA and ICARS scores, but not age, AAO, DD, or number of CAG repeats, are significant predictors of serum CHIP levels. Ataxia rating scales are widely used to evaluate cerebellar diseases. SARA and ICARS are thought to be the most reliable and valid scores for the severity of SCA3 (23). Earlier studies have shown that age-adjusted SARA and ICARS scores were directly correlated with the number of CAG repeats in patients with SCA3 (24). This study found that CHIP levels were associated with the progression of SCA3, thus, providing further support for the potential use of CHIP as a biomarker. Considering the convenience and safety of serum collection, its use is preferable to that of CSF.

We also found that serum levels of CHIP are positively correlated with age, but not significantly correlated with sex in healthy controls. Aging is accompanied by the progressive accumulation of misfolded proteins and genetic damage (25, 26). As a key component of the PQC system, CHIP may regulate the degradation of aggregates. Furthermore, CHIP could stabilize SirT6, a protein that is involved in DNA repair and metabolism, and promote DNA repair (27). Thus, age changes may partially account for increases in the protein expression in both groups.

This study has several limitations. First, as we designed an exploratory case-control study with no primary outcomes, no sample size calculation was performed. Second, the relationships between CSF CHIP levels and other factors were not analyzed due to the limited sample size. The comparison of CSF and serum CHIP levels may also be affected by this. Finally, while our study compared CHIP concentrations between patients with SCA3 and healthy controls, the change in CHIP levels over time remains unknown. We used a variety of methods to control bias. For example, the neurological assessment was performed independently by two neurologists, and each sample was measured twice on the same plate. However, since the assessments of the disease scales were subjective, bias may not have been completely eliminated. Prospective longitudinal studies are required to elucidate the variations in CHIP levels.

While SCA3 can only be diagnosed by genetic tests, structural and functional magnetic resonance imaging abnormalities, aberrant electrophysiology, and altered glucose utilization are also associated with SCA3 (28). However, convenient and objective methods for evaluating the severity of this disease are lacking. Molecular biomarkers are expected to be sensitive and specific in the staging and prognosis of the disease. In this study, we first determined the concentrations of CHIP in the CSF and sera of patients with SCA3 and healthy controls. Serum CHIP levels were associated with SARA and ICARS scores, which reflect the severity of the disease. Future studies with larger populations are needed to confirm our findings. The role of CHIP in the progression of SCA3 also requires further investigation.

## Data Availability Statement

The raw data supporting the conclusions of this manuscript will be made available by the authors, without undue reservation, to any qualified researcher.

## Ethics Statement

This study was approved by the Ethics Review Committee of Zhengzhou University, and informed consent was obtained from all subjects. This study did not interrupt any part of diagnosis or treatment, and all the clinical data would be proper preservation.

## Author Contributions

YX and CS designed the whole work. ZH and ZY did the experiment. ZH analyzed the data and wrote the paper. SZ and ZY recruited patients. ZH, SZ, and QZ collected samples and completed genotype identification. YL, JY, and YW assessed the clinic scales of patients. CM polished the article.

### Conflict of Interest

The authors declare that the research was conducted in the absence of any commercial or financial relationships that could be construed as a potential conflict of interest.
